# Metabolomic Profiling of Human Urine Related to Mycotoxin Exposure

**DOI:** 10.3390/toxins17020075

**Published:** 2025-02-08

**Authors:** Nuria Dasí-Navarro, Sonia Lombardi, Pilar Vila-Donat, Sabrina Llop, Jesus Vioque, Raquel Soler-Blasco, Ana Esplugues, Lara Manyes, Manuel Lozano

**Affiliations:** 1 Biotech Agrifood, Faculty of Pharmacy and Food Sciences, University of Valencia, 46100 Burjassot, Spain; nuria.dasi@uv.es (N.D.-N.); pilar.vila@uv.es (P.V.-D.); manuel.lozano@uv.es (M.L.); 2 Department of Pharmacy, University of Naples Federico II, Via Domenico Montesano 49, 80131 Naples, Italy; sonia.lombardi@unina.it; 3 Epidemiology and Environmental Health Joint Research Unit, FISABIO—Universitat Jaume I—Universitat de València, 46020 València, Spain; sabrina.llop@fisabio.es (S.L.); raquel.soler@uv.es (R.S.-B.); ana.esplugues@uv.es (A.E.); 4 Spanish Consortium for Research on Epidemiology and Public Health (CIBERESP), 28029 Madrid, Spain; vioque@umh.es; 5 Alicante Institute of Health and Biomedical Research, University Miguel Hernandez (ISABIAL-UMH), 03010 Alicante, Spain; 6 Department of Nursing, Faculty of Nursing and Podiatry, University of Valencia, 46010 Valencia, Spain

**Keywords:** biomonitoring, mycotoxins, metabolomics, urine, HPLC-Q-TOF-MS

## Abstract

Human exposure to mycotoxins is a global concern since several mycotoxins, such as enniatins and aflatoxins, have shown carcinogenic and neurotoxic effects, and the toxicologic mechanisms of most of them still need to be clarified. This study aims to investigate the metabolic pathways affected by mycotoxin exposure by evaluating metabolite alterations in urine. The participants were 540 women from the Spanish Childhood and Environment Project (INMA). For metabolite identification, a dilute and shoot extraction, followed by HPLC-Q-TOF-MS identification analysis, was performed. Data were processed using Agilent Mass Hunter Workstation with the METLIN database, Agilent Mass Profiler Professional 10.0, and Metaboanalyst 6.0. Over 2000 metabolites were obtained in each sample after feature extraction, and the most significant metabolites (*p*-value ≤ 0.05, fold change ≥ 2.0) were considered for pathway analysis. Enrichment analysis and topology showed that the most significantly affected pathway was the biosynthesis of unsaturated fatty acids (adjusted *p*-value = 0.007), with four metabolomic hits associated: linoleic acid, octadecanoic acid/stearic acid, an arachidonic acid metabolite, and (9Z)-octadecenoic acid/oleic acid. Other related pathways (unadjusted *p*-value ≤ 0.1) included fatty acid biosynthesis, glycerophospholipid metabolism, and ether lipid metabolism. The present study highlights the importance of metabolomics in increasing knowledge of the toxicity mechanisms and health effects of mycotoxins, especially emerging ones.

## 1. Introduction

Mycotoxin exposure occurs daily through food consumption and other environmental factors; therefore, multi-mycotoxin detection methods, including those for emerging mycotoxins, are important tools for assessing general exposure in human biomonitoring [1]. Nonetheless, exposome-based approaches that include mycotoxins are increasingly necessary to provide a broader perspective on the associations with other external factors [2]. Furthermore, recent studies are investigating the associations between mycotoxin exposure and dietetic and sociodemographic factors, showing that these variables influence mycotoxin content [3,4,5].

Different mechanisms for the toxicity of various mycotoxins have been proposed. The literature shows that enniatin (ENN) toxicity is related to their ionophore properties, which affect cell homeostasis, the induction of apoptosis, and the production of reactive oxygen species (ROS) [6,7]. In contrast, aflatoxin B1 (AFB1) is metabolized in the liver by the P450 enzyme system into the ultimate carcinogen AFB1-8,9-epoxide (AFBO), making its carcinogenic effect well known, as well as its negative effect on nutritive status, growth/development, and immune system function [8]. Nevertheless, transcriptional in vitro analysis on human epithelial cells ECV 304 showed that ENNs and AFs could penetrate the blood–brain barrier [9]. Compromise of the blood–brain barrier by mycotoxins can produce oxidative stress and neuroinflammation, leading to the apoptosis of brain cells [10]. Moreover, the cytotoxic effects of ENNs A1 and B1 were tested in SH-SY5Y human neuroblastoma cells, revealing that cell viability was reduced due to caspase-dependent apoptosis induction, ROS production, and alterations in toxin Ca^2+^ homeostasis [11]. Quantitative structure–activity relationship (QSAR) models have been employed to investigate the chemo-mathematical patterns associated with mycotoxins that induce lipid peroxidation, which affects cell membrane integrity and is related to oxidative stress, inflammation, mitochondrial dysfunction, and can lead to neurodegenerative diseases [12].

Studies on the toxicity mechanisms of emerging mycotoxins are scarce; therefore, a further step in mycotoxin research is metabolomics, which is a pioneering technique in the omics sciences that is useful for assessing internal and complex environmental exposures [13]. Metabolomics has emerged as a powerful tool for identifying novel biomarkers of contaminants, as well as for exploring toxicity mechanisms and metabolic interactions [14].

In animal fluids, such as urine and plasma, metabolomics has been employed previously to study changes in urine composition related to mycotoxin-controlled exposures to study metabolic perturbations associated with metabolic pathway alterations [15]. Regarding studies involving human samples, most metabolomic studies have been employed to investigate changes in urine composition related to dietary habits and lifestyle, as well as diseases like obesity [16]. The impact of environmental stress or altered lifestyle and diet has also been studied [17]. Metabolomic studies in humans related to chemical contaminants have primarily focused on individuals occupationally exposed to pesticides, analyzing plasma and urine samples. These studies have identified disturbances in several metabolic pathways, including lipid and amino acid metabolism [14], as well as environmental pollutant exposure-related biomarkers in urine associated with metabolic pathways involved in amino acid, lipid, arachidonic acid, and organic acid metabolism [18].

Several studies have related mycotoxins to these metabolic changes. A study using a 3D HepaRG human hepatocyte spheroid model was performed to investigate ENN and beauvericin (BEA) toxicity mechanisms, showing that ENNs and BEA significantly altered lipid metabolism pathways, specifically those involving glycerophospholipids and sphingolipids, as well as other metabolic pathways, such as linoleic acid metabolism, α-linolenic acid metabolism, arachidonic acid metabolism, and glycosylphosphatidylinositol (GPI)-anchor biosynthesis [19]. Another study carried out on the serum and urine of weaned rabbits showed that the mycotoxin deoxynivalenol (DON) induced metabolites that affected several pathways, like glycerophospholipid metabolism, tryptophan metabolism, pentose and glucuronate interconversions, ether lipid metabolism, and glycine, serine, and threonine metabolism [20]. In an in vivo study using urine and serum from Winstar rats exposed to the mycotoxin patulin (PAT), alterations in key metabolic pathways related to amino acids and fatty acids were found [15].

This work aims to investigate the metabolic pathways affected by mycotoxin exposure by evaluating the human urine metabolome to provide more data on the combined toxic effects of mycotoxins.

## 2. Results

**Figure 1:**
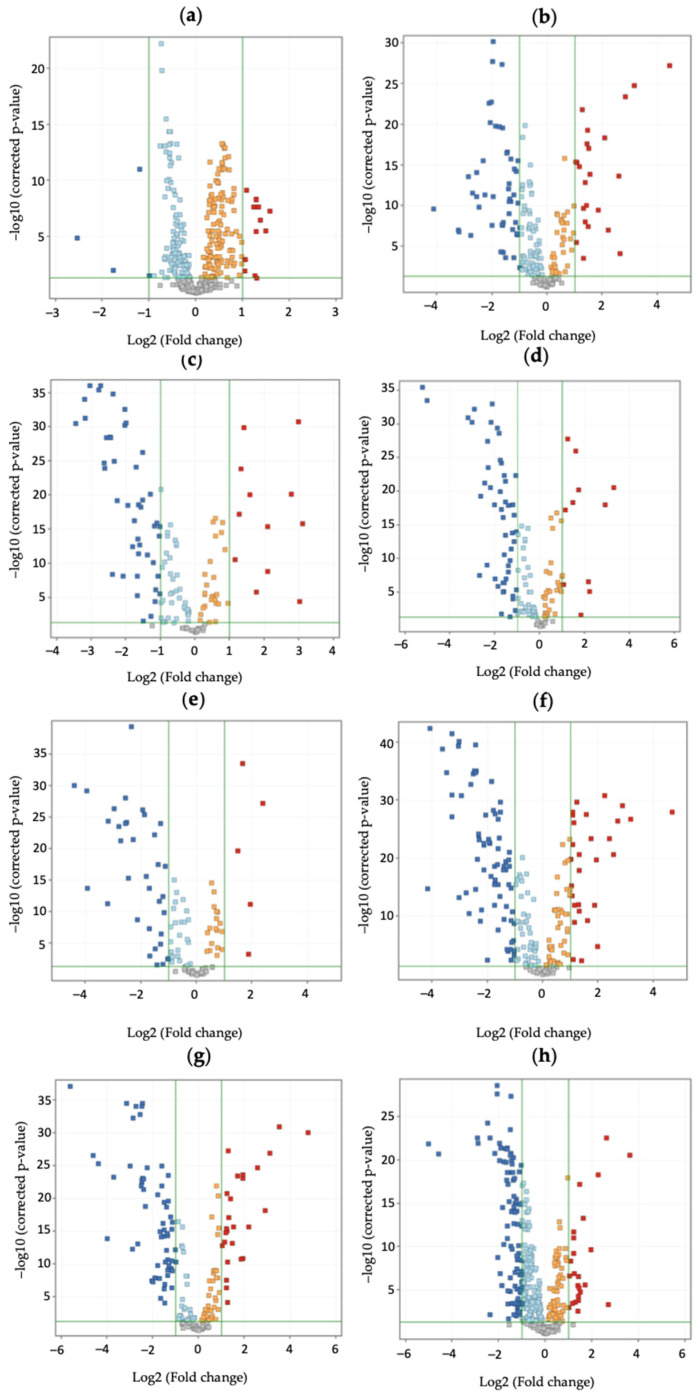
Volcano plots for each *t*-test (Y-axis indicates the corrected *p*-value, and X-axis indicates the log2 fold change) performed between positive sample batches and control, clustered according to crescent sum of enniatin concentration, (**a**) 0.4–2.1 ng/mg; (**b**) 2.2–2.7 ng/mg; (**c**) 2.8–3.37 ng/mg; (**d**) 3.4–3.97 ng/mg; (**e**) 4.0–5.17 ng/mg, (**f**) 5.2–7.47 ng/mg; (**g**) 7.5–52.1 ng/mg, and the sum of aflatoxin concentration, (**h**) 0.6–9.1 ng/mg. Blue dots represent significantly downregulated metabolites, red dots represent significantly upregulated metabolites, and gray dots represent non-significantly changed metabolites.

Over 2000 molecular features were obtained in each sample after data processing with the MassHunter Workstation software, version 10.0 (Agilent Technologies, Santa Clara, CA, USA) and up to 175 significant metabolites (*p*-value ≤ 0.05, |log2FC| ≥ 2.0) were identified across all samples (Table S1) as candidates for pathway analysis, of which 41 were upregulated and 134 were downregulated. Figure 1 shows the volcano plots for each analyzed batch of samples according to their level of quantified mycotoxin exposure, selecting the metabolites that meet the stated thresholds for log2FC and adjusted *p*-values.

**Figure 2:**
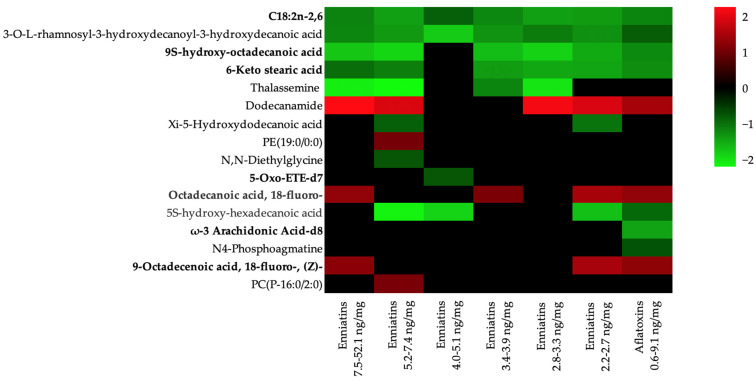
Heatmap representation of metabolites involved in affected pathways. Green and red represent significant increasing or decreasing fold change values of metabolites, respectively. The scale displays the fold change.

Up to 27 affected pathways were identified (Table S2). Among these 27 pathways, the biosynthesis of unsaturated fatty acids pathway was the most significant (Holm adjusted *p*-value < 0.001 and false discovery rate (FDR) = 0.007), with four metabolomic hits associated: linoleic acid, octadecanoic acid/stearic acid, arachidonic acid metabolite, and (9Z)-octadecenoic acid/oleic acid (Table 1). Other related pathways with metabolites that obtained an unadjusted *p*-value ≤ 0.1 were glycerophospholipid metabolism (0.012), fatty acid biosynthesis (0.026), ether lipid metabolism (0.030), linoleic acid metabolism (0.068), glycine, serine, and threonine metabolism (0.076), and arginine and proline metabolism (0.089) (Table S2). Table 1 shows the seven interrelated pathways affecting up to 16 different metabolites, 4 of which were upregulated and 11 were downregulated after exposure to both ENNs and AFs. Figure 2 shows a heatmap of the metabolites involved in the pathways affected by mycotoxin concentrations, with adjusted *p*-values ≤ 0.05. Upregulated metabolites included dodecanamide, PE(19:0/0:0), octadecanoic acid, 18-fluoro-, 9-octadecenoic acid, 18-fluoro-, (Z)- and PC(P-16:0/2:0); downregulated metabolites included C18:2n-2,6, 3-O-L-rhamnosyl-3-hydroxydecanoyl-3-hydroxydecanoic acid, 9S-hydroxy-octadecanoic acid, 6-keto stearic acid, thalassemine, xi-5-hydroxydodecanoic acid, N,N-diethylglycine, 5-oxo-ETE-d7, 5S-hydroxy-hexadecanoic acid, ω-3 arachidonic acid-d8, and N4-phosphoagmatine. Some metabolites were consistently downregulated across almost all mycotoxin concentration batches, specifically C18:2n-2,6, 3-O-L-rhamnosyl-3-hydroxydecanoyl-3-hydroxydecanoic acid, 9S-hydroxy-octadecanoic acid, and 6-keto stearic acid. However, dodecanamide was the metabolite with the most upregulated presence among the batches, followed by octadecanoic acid and 18-fluoro-. Regarding mycotoxin differences, only two metabolites, ω-3 arachidonic acid-d8 and N4-phosphoagmatine, were found solely in the alfatoxin batch.

**Figure 3:**
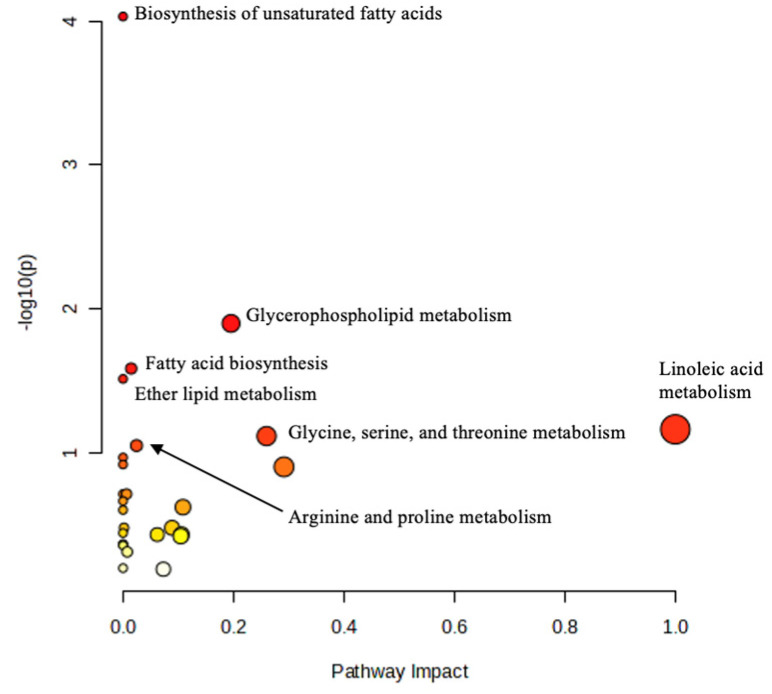
Topology of the influence of enniatin and aflatoxin oral exposure on pathways related to differentially expressed urine metabolites.

These metabolites affected up to seven pathways (Table 1), with the biosynthesis of unsaturated fatty acids being the most significantly affected (*p*-value < 0.001). Each pathway had some related metabolomic hits, which were matched with identified metabolites to standardize compound labels contained in the pathway library (*Homo sapiens* (human), to Metaboanalyst software, version 6.0 (https://www.metaboanalyst.ca/). The affected pathways and associated hits included the biosynthesis of unsaturated fatty acids (linoleic acid, octadecanoic acid/stearic acid, arachidonic acid metabolite, ((9Z)-octadecenoic acid/oleic acid), fatty acid biosynthesis (decanoic acid, dodecanoic acid, and hexadecanoic acid), glycerophospholipid metabolism (glycerophosphoethanolamine), ether lipid metabolism (glycerophosphoethanolamine and glycerophosphocholine), linoleic acid metabolism (linoleic acid), glycine, serine, and threonine metabolism (phosphagen/creatine and glycine), and arginine and proline metabolism (phosphagen/creatine and agmatine). The topology of the influence of ENNs and AFs on these pathways is shown in Figure 3.

## 3. Discussion

The simultaneous presence of regulated and non-regulated mycotoxins in food products is common since one fungus can produce more than one type [21], leading to co-exposure, with metabolites found in human samples such as urine [5,22]. Multi-mycotoxin studies show a high prevalence of emerging mycotoxins, such as ENNs, which are non-regulated contaminants whose toxicokinetic data are still scarce [7]. The toxic effects of AFs are well known, especially the carcinogenic effect of AFB1, and despite being regulated, food exposure is still a concern, especially in Africa and Asia [8]. However, data on combined cytotoxic effects are still scarce, but some in vitro studies have demonstrated significant toxicity in various cell lines in combinations of enniatin B (ENNB), sterigmatocystin, and AFB1 in Caco-2, HepG2, and Hek-293 cells [23]. In the present study, metabolic disturbances caused by oral exposure to different concentrations of ENNs and AFs in women’s urine were analyzed, showing an association with lipid metabolism pathways. Several in vitro studies have revealed that lipid metabolism disruption is related, among other effects, to neurotoxicity [10,24,25].

### 3.1. Lipid Metabolism

Lipids are essential for supporting the normal function of brain tissue, and their disruption mainly involves sphingolipid metabolism and fatty acid degradation pathways, which are related to the neurotoxicity of AFB1 [24]. Emerging mycotoxins, such as enniatin A (ENNA) and ENNB, were tested in SH-SY5Y human neuroblastoma cells, and effects on mitochondrial membrane potential were observed, leading to cell apoptosis [25].

In the present study, the biosynthesis of unsaturated fatty acids was the most significantly affected pathway (*p*-value < 0.001), with five downregulated metabolites (C18:2n-2,6, 9S-hydroxy-octadecanoic acid, 6-keto stearic acid, 5-oxo-ETE-d7, and ω-3 arachidonic acid-d8) and two upregulated metabolites (octadecanoic acid, 18-fluoro-, 9-octadecenoic acid, and 18-fluoro-, (Z)-) involved. Other affected pathways included fatty acid biosynthesis, which had three downregulated metabolites (3-O-L-rhamnosyl-3-hydroxydecanoyl-3-hydroxydecanoic acid, xi-5-hydroxydodecanoic acid, and 5S-hydroxy-hexadecanoic acid) and one upregulated metabolite (dodecanamide); glycerophospholipid metabolism, with one upregulated metabolite (PE(19:0/0:0)); and ether lipid metabolism, with two upregulated metabolites (PE(19:0/0:0) and PC(P-16:0/2:0)). Similar results were observed in current studies, showing that mycotoxin exposure altered lipid metabolism pathways, specifically those involving glycerophospholipids and sphingolipids [19], as well as tryptophan metabolism, pentose and glucuronate interconversions, ether lipid metabolism, and glycine, serine, and threonine metabolism [20].

Hepatocyte exposure to ENNB and BEA was investigated, and a pathway enrichment analysis showed that ENNB affected lipid membranes, enriched glycerophospholipid metabolism, and upregulated the gene for lipoprotein lipase, which breaks down lipoproteins into free fatty acids. The RNA sequencing analysis indicated that BEA affected glutathione metabolism, ENNB affected the amino acid synthesis of glutathione precursors (i.e., glycine and cysteine), and both mycotoxins enriched the ferroptosis pathway [26]. In neuronal cells, T-2 toxin induced oxidative stress responses related to neurotoxicity, such as ROS generation and lipid peroxidation, by crossing the blood–brain barrier by altering permeability [27].

### 3.2. Linoleic Acid Metabolism

Linoleic acid (C18:2 n-6) is known as an essential n-6 polyunsaturated fatty acid (PUFA) and a precursor converted to arachidonic acid (C20:4 n-6). The central nervous system is composed of phospholipids, glycolipids, cholesterol, triglycerides, and cholesterol esters. A total of 30% of the total fatty acids in the brain are PUFAs, including docosahexaenoic acid (DHA) and arachidonic acid, which are enriched in brain membrane phospholipids and are involved in diverse brain pathologies. It has been shown that linoleic acid influences multiple cellular responses, such as oxidative stress, inflammation, and mitochondrial function [28].

In the current study, the linoleic acid metabolism pathway was affected by one downregulated metabolite involved, C18:2 n-2,6. This is in accordance with results obtained from a human 3D HepaRG spheroid model, in which the hepatotoxic effects of ENNs and beauvercin were studied, showing alterations in lipid metabolism pathways, specifically glycerophospholipid and sphingolipid metabolism pathways, by disturbing intracellular lipid homeostasis in hepatocytes, as well as in other pathways, such as linoleic acid metabolism, α-linolenic acid metabolism, arachidonic acid metabolism, and glycosylphosphatidylinositol (GPI)-anchor biosynthesis [19].

### 3.3. Glycine, Serine, and Threonine Metabolism

Threonine is an essential amino acid and a precursor for glycine synthesis, whose deficiency in mammals has been associated with depression and neurological dysfunction [29]. Serine and glycine contribute to the formation of proteins, nucleic acids, and lipids, and serine biosynthesis is related to cellular antioxidant capacity [30].

In the present work, the glycine, serine, and threonine metabolism pathway was affected by two downregulated metabolites, thalassemine and N,N-diethylglycine. The toxic effects of DON were analyzed in the serum and urine of weaned rabbits using LC–MS/MS metabolomics, showing biomarkers in serum mainly involved in glycerophospholipid metabolism, tryptophan metabolism, and pentose and glucuronate interconversions, while those in urine samples were involved in caffeine metabolism, glycine, serine and threonine metabolism, and terpenoid backbone biosynthesis [20].

### 3.4. Arginine and Proline Metabolism

Arginine is a conditionally essential amino acid implicated in polyamine synthesis and proline synthesis and is the only biosynthetic substrate for nitric oxide [31]. Proline is a secondary amino acid involved in protein biosynthesis and is related to osmoregulation, stress protection, and cellular signaling processes [32].

A current study showed that the arginine and proline metabolism pathway was affected by two downregulated metabolites, thalassemine and N4-phosphoagmatine. In an in vivo study of mice exposed to different doses of PAT, transcriptional and metabolic results in the small intestine and colon showed that seven metabolic pathways were affected in both organs, including glutathione metabolism, arginine biosynthesis, and arginine and proline metabolism [33]. No further investigations have been found linking arginine and proline metabolism alterations with mycotoxins in humans; however, urinary metabolomics analysis was carried out in pregnant women to assess the combined effects of other environmental contaminants (Zn and Cd), revealing that the arginine and proline pathways were upregulated [34].

### 3.5. Strengths and Limitations

There is scarce research on metabolomics and mycotoxin exposure, especially focusing on the female population. However, this study has some limitations; around 30% of the initial participants were not included in this study due to the characteristics of interest sought in the samples. Mycotoxins were analyzed in single-spot urine samples, which lack long-term metabolic changes. Participants’ dietary habits and lifestyle could influence metabolite results; however, this could be addressed in further research. The major strength is the inclusion of emerging and legislated mycotoxins for co-exposure effects. This cohort is part of a larger Spanish study involving other cohorts and more biological samples, resulting in a substantial database of sociodemographic, lifestyle, and dietary data.

## 4. Conclusions

To the best of our knowledge, this is the first untargeted metabolomic study of human urine samples that associates metabolic alterations with combined exposure to emerging and legislated mycotoxins. Our results showed that exposure to ENNs and AFs was significantly related to lipid metabolism pathways, which are constituents of biological membranes, specifically unsaturated fatty acid biosynthesis, general fatty acid biosynthesis, glycerophospholipid metabolism, and ether lipid metabolism. Further research on the combined exposure to emerging mycotoxins is needed to better understand their toxicity mechanisms and improve risk assessment. Increasing knowledge of the effects of emerging mycotoxins on the metabolomic profile allows for the development of new health and mitigation strategies and consideration of their inclusion in food safety regulations.

## 5. Materials and Methods

### 5.1. Study Population and Design

Urine samples belong to the participants in the INMA (Environment and Childhood project, https:www.proyectoinma.org) project, which is a multicenter birth cohort study conducted in various geographical areas of Spain assessing exposure to environmental pollutants from air, water, and diet during pregnancy and childhood and the effects on child and adolescent growth, health, and development [35]. During the first antenatal visit at La Fe Hospital in Valencia (Spain), women were recruited (*n* = 855). The criteria for inclusion included residing in the study area, being at least 16 years old, having a singleton pregnancy, not having followed any assisted reproduction program, wanting to deliver at the reference hospital, and not having communication difficulties. Their children were enrolled at birth and monitored over the years. For the current study, samples were collected from mothers 4 years after childbirth, with 524 analyzed samples included in a previous study in which mycotoxin presence was evaluated; only urine samples with quantified mycotoxins were included in the metabolomic study (*n* = 151) [5].

Informed consent was obtained from all participants in each phase, and the study protocol was approved by the Public Health Research Centre in Valencia (CSISP) and La Fe Hospital Ethics Committees.

### 5.2. Sample Extraction Essays

Depending on the concentration of mycotoxins previously quantified and detected in a preceding article [5], 151 samples in which ENNs and AFs were quantified were selected. The samples were divided into 8 batches, 7 of which had increasing concentrations of quantified ENNs: 0.4–2.1; 2.2–2.7; 2.8–3.3; 3.4–3.9; 4.0–5.1; 5.2–7.4; and 7.5–52.1 ng/mg, and 1 one of which had quantified AFs at 0.6–9.1 ng/mg, plus a control batch containing 20 negative samples without mycotoxin detection. In brief, after thawing the urine, samples were diluted with Milli-QSP® Reagent Water System (Millipore, Bedford, MA, USA) to normalize samples to concentrations of 0.1 mg/mL creatinine. Afterward, to remove solid components, the urine samples were centrifuged for 10 min at 25 830 g and 4 °C. Subsequently, supernatants were collected, and a 1:5 dilution was carried out before the LC-Q-TOF-MS analysis.

Throughout all analyses, the stability and efficiency of the system were assessed employing two distinct biological sample types. These included long-term reference samples consisting of a pool of urine samples from healthy women with different conditions from the study population and batch-specific quality control samples (QCs), which were prepared by mixing equal volumes (75 µL) of all the study samples included in each batch. Unlike QCs, reference samples were not biologically related to the samples under study, making them not directly representative of the sample population. Detailed information about the mycotoxin extraction procedure can be found elsewhere [5,36].

### 5.3. LC-Q-TOF-MS Analysis

6540 Ultra-High Definition (UHD) Accurate Mass Quadrupole time of flight (Q-TOF) spectrometer coupled with an Agilent 1260 HPLC system, and a Jet Stream dual Electrospray ionization (Dual AJS ESI) interface (Agilent Technologies, Santa Clara, CA, USA) was used for the spectrometric analysis. Metabolites were separated using a reverse phase C18 column (Zorbax Eclipse Plus, 2.1 mm × 150 mm, 3.5 μm, Agilent Technologies, Santa Clara, CA, USA) with an injection of 3 μL. The eluent phases included Phase A (water with 0.1% formic acid *v*/*v*) (Acros organics, Geel, Belgium) and Phase B (Methanol, Fisher Scientific (Loughborough, UK)), and a flow of 0.4 mL/min was used. To achieve an effective separation, the following gradient was used: initially 5% B and increased to 30% B after 30 min until arriving at 100% B in 10 min. After 10 min, it was linearly decreased to 5% B, and then this gradient was held for 10 min. The column and autosampler compartment temperatures were set at 25 and 4 °C, respectively.

Detection was performed in positive-ion mode over a range of 50 to 1700 *m*/*z*. Ultra-high pure nitrogen was used as a drying and nebulizer gas at temperatures of 200 and 350 °C and flows of 10 and 12 L/min, respectively. Other optimized parameters were as follows: capillary voltage, 4000 V; nebulizer, 20 psi; fragmentor, 130 V; nozzle voltage, 500 V; skimmer, 45 V; and octopole, 1 RF Vpp, 750 V.

Samples were analyzed as follows: 1 blank, 1 long-term reference sample, 1 QC sample, 20 samples alternated by 1 blank every 5 samples, at the end, another QC sample, 1 long-term reference sample, and finally, 1 blank. Samples were clustered in batches of 20 samples, depending on the sum of the ENN concentration (ng/mg) and the sum of the AF concentration (ng/mg). Finally, an MS/MS analysis of the QC sample was performed to facilitate the identification of potential biomarkers. This experiment was performed using nitrogen as the collision gas with the following collision energy values: 10 eV, 20 eV, and 40 eV.

### 5.4. Data Processing

Chemical features found in the acquired data were extracted and integrated using Mass Hunter Workstation software, version 10.0 (Agilent Technologies, Santa Clara, CA, USA), which provides untargeted molecular feature extraction using the METLIN database [37] to identify features as compounds and also visualizes extracted ion chromatograms and mass spectral data associated with each feature.

### 5.5. Statistical Analysis

A significance testing and a fold change analysis was performed using Mass Profiler Professional, version 15.0 (Agilent Technologies, Santa Clara, CA, USA). Due to the large number of samples, the chosen strategy was to carry out a *t*-test between each sample batch and the control. Molecular features were filtered by abundance (minimum absolute abundance of 5000 counts), mass range (50–1000), number of ions per feature (minimum of 2), and charge state in order to reject low-intensity data. Filtering by maximum mass allows us to reject non-significant masses. Subsequently, features were aligned across samples based on tolerances established by retention time (RT) and mass (RT Window: 0.1% and 0.15 min) and then normalized to their selected statistical abundance across all samples. Finally, significance testing and a fold change analysis were performed (*p*-value ≤ 0.05, log2 fold change (log2FC) ≥ 2.0), which provides quality control for the analysis and improves results, leading to a list of altered metabolites that are either down- or upregulated regarding both the sum of the ENN concentration (ng/mg) and the sum of the AF concentration (ng/mg).

### 5.6. Metabolite Identification and Metabolite Pathway Analysis

In order to standardize the compound labels, the obtained metabolites were matched with compounds present in databases such as Pubchem, the Human Metabolome Database (HMDB), Chemical Entities of Biological Interest (CHEBI), and the Kyoto Encyclopedia of Genes and Genomes (KEGG) [38,39,40,41] to compile a list of annotated metabolites present across all batches. This compound list was imported to Metaboanalyst software, version 6.0 (https://www.metaboanalyst.ca/) [42] for a pathway enrichment analysis and topology, selecting a hypergeometric test as the over-representation analysis method. A statistical significance level of <0.1 was considered to select interrelated pathways.

## Figures and Tables

**Table 1:** Pathway enrichment analysis results.

Pathway	Hit	Metabolite	Enniatins 2.2–52.1 ng/mg (Log FC)	Aflatoxins 0.6–9.1 ng/mg (Log FC)	Regulation	Pathway Unadjusted *p*-Value	Pathway FDR
Biosynthesis of unsaturated fatty acids	Linoleic acid	C18:2n-2,6	−1.7 ± 0.3	−1.6 ± 0.0	Down	<0.001	0.007
	Octadecanoic acid/stearic acid	9S-hydroxy-octadecanoic acid	−2.4 ± 0.2	−1.7 ± 0.0	Down	<0.001	0.007
	Octadecanoic acid/stearic acid	Octadecanoic acid, 18-fluoro-	1.2 ± 0.2	1.2 ± 0.0	Up	<0.001	0.007
	Octadecanoic acid/stearic acid	6-Keto stearic acid	−1.8 ± 0.3	−1.7 ± 0.0	Down	<0.001	0.007
	Arachidonic acid metabolite	5-Oxo-ETE-d7	−1.0 ± 0.0	−	Down	<0.001	0.007
	Arachidonic acid metabolite	ω-3 Arachidonic Acid-d8	−	−2.0 ± 0.0	Down	<0.001	0.007
	(9Z)-Octadecenoic acid/Oleic acid	9-Octadecenoic acid, 18-fluoro-, (Z)-	1.2 ± 0.2	1.2 ± 0.0	Up	<0.001	
Fatty acid biosynthesis	Decanoic acid	3-O-L-rhamnosyl-3-hydroxydecanoyl-3-hydroxydecanoic acid	−1.8 ± 0.3	−1.1 ± 0.0	Down	0.025	0.608
	Dodecanoic acid	Dodecanamide	2.0 ± 0.2	1.4 ± 0.0	Up	0.025	0.608
	Dodecanoic acid	Xi-5-Hydroxydodecanoic acid	−1.3 ± 0.2	−	Down	0.025	0.608
	Hexadecanoic acid	5S-hydroxy-hexadecanoic acid	−2.6 ± 0.3	−1.3 ± 0.0	Down	0.025	0.608
Glycerophospholipid metabolism	Glycerophosphoethanolamine	PE(19:0/0:0)	1.0 ± 0.0	−	Up	0.012	0.501
Ether lipid metabolism	Glycerophosphoethanolamine	PE(19:0/0:0)	1.0 ± 0.0	−	Up	0.030	0.608
	Glycerophosphocholine	PC(P-16:0/2:0)	1.0 ± 0.0	−	Up	0.030	0.608
Linoleic acid metabolism	Linoleic acid	C18:2n-2,6	−1.7 ± 0.3	−1.6 ± 0.0	Down	0.068	0.991
Glycine, serine, and threonine metabolism	Phosphagen/creatine	Thalassemine	−2.6 ± 0.7		Down	0.076	0.991
	Glycine	N,N-Diethylglycine	−1.0 ± 0.0	−	Down	0.076	0.991
Arginine and proline metabolism	Phosphagen/creatine	Thalassemine	−2.6 ± 0.7		Down	0.089	0.991
	Agmatine	N4-Phosphoagmatine	−	−1.0 ± 0.0	Down	0.089	0.991

## Data Availability

The original contributions presented in this study are included in the article and Supplementary Materials. Further inquiries can be directed to the corresponding author.

## Supplementary Materials

The following supporting information can be downloaded at https://www.mdpi.com/article/10.3390/toxins17020075/s1: Table S1. Metabolite identification from METLIN database and statistical analysis; Table S2. Results from pathway analysis.

## Abbreviations

The following abbreviations are used in this manuscript:

AFs	Aflatoxins
AFB1	Aflatoxin B1
AFBO	Aflatoxin B1-8,9-epoxide
BEA	Beauvericin
CHEBI	Chemical Entities of Biological Interest
DON	Deoxynivalenol
ENNs	Enniatins
ENNA	Enniatin A
ENNB	Enniatin B
ESI	Electrospray ionization
HMDB	Human Metabolome Database
HPLC	High performance liquid
KEGG	Kyoto Encyclopedia of Genes and Genomes
LC	Liquid chromatography
OTα	Ochratoxin alpha
PAT	Patulin
PUFA	Polyunsaturated fatty acid
QCs	Quality control samples
QSAR	Quantitative structure–activity relationship
Q-TOF	Quadrupole time of flight
RNA	Ribonucleic acid
ROS	Reactive oxygen species
UHD	Ultra-High Definition
