# Supplementary material for: Metabolomic Profiling of Human Urine Related to Mycotoxin Exposure
Source: Toxins (Basel). 2025 Feb 8;17(2):75. doi: 10.3390/toxins17020075 (PMC11860571; doi:10.3390/toxins17020075)
Supplement: Supplementary file 1 [file toxins-17-00075-s001.zip › toxins-3437554-supplementary.pdf]

Table S1. Metabolites identification from METLIN database and statistical analysis.

Metabolite name	SumaENNs 0.4-52.1 ng/mg (Log 2FC)	SumAFs 0.6-9.1 ng/mg (Log2FC)	Regulation
4-keto palmitic acid	-2,68		Downregulated
PGF1β	-2,33	-1,61	Downregulated
8-hydroxy-9,11-octadecadiynoic acid	-2,26		Downregulated
1-Oleoyl-2-acetyl-sn-glycerol	1,47		Upregulated
Coformycin	-1,62	-1,22	Downregulated
9,10-12,13-Diepoxyoctadecanoate	-1,55	-1,03	Downregulated
N-hydroxy arachidonoyl amine	2,61	1,95	Upregulated
6Z,9Z-Heneicosadiene	1,81		Upregulated
C18:2n-2,6/Linoleic acid	-1,70	-1,62	Downregulated
3-O-L-rhamnosyl-3-hydroxydecanoyl-3-hydroxydecanoic acid	-1,84	-1,14	Downregulated
3'-Hydroxytrimethoprim	-3,07		Downregulated
Theaspirone A	-1,29		Downregulated
Polystachin (flavone)	-1,11		Downregulated
9S-hydroxy-octadecanoic acid	-2,39	-1,68	Downregulated
6-Keto stearic acid	-1,76	-1,72	Downregulated
Riesling acetal	-1,38	-1,17	Downregulated
8,8-Diethoxy-2,6-dimethyl-2-octanol	-1,60		Downregulated
Cis-3-Hexenyl hexanoate	-1,49		Downregulated
Glutaral (Glutaraldehyde)	-2,41	-1,62	Downregulated
PGF2α Alcohol methyl ether	1,38		Upregulated
Disopyramide	-2,47		Downregulated

3-(Pyrazol-1-yl)-L-alanine	-1,55		Downregulated
N-Ethyl-N-methylcathinone	1,63		Upregulated
Leu Leu Arg (Oligopeptide)	-2,33	-1,66	Downregulated
Tebutam (Imidacloprid)	-3,84		Downregulated
(-)-trans-Carveol glucoside	-1,27		Downregulated
Thalassemine (Phosphagen)	-2,59		Downregulated
Sintaxanthin	-1,63		Downregulated
PE(22:2(13Z,16Z)/12:0)	1,25		Upregulated
3-Butylidene-7-hydroxyphthalide	-1,43		Downregulated
Cuscohygrine	1,15		Upregulated
1,2,3-Tris(1-ethoxyethoxy)propane	-2,20		Downregulated
Arg Ser Ser (Oligopeptide)	-2,11		Downregulated
JWH 018 N-pentanoic acid metabolite-d4	-2,29		Downregulated
Lys Asn Asp (Oligopeptide)	-2,27		Downregulated
Dodecanamide	1,99	1,42	Upregulated
7Z-Tetradecenal	2,95	2,27	Upregulated
5-Amino-4-imidazole carboxylate	-1,43		Downregulated
4-tert-Octylphenol	-1,48	-1,24	Downregulated
6-Phenyl-3-hexen-2-one	2,12	1,39	Upregulated
Nonic acid	-1,25	-1,64	Downregulated
(10S)-Juvenile hormone III acid diol	-3,55		Downregulated
Globulol	-2,21	-1,05	Downregulated
1-Phenyl-2-(diethylamino)-1-propanol	1,09		Upregulated
Quinacetol (Ketone)	-1,24	-1,10	Downregulated
Xi-5-Hydroxydodecanoic acid	-1,28		Downregulated

Dihydrodeoxy-8-epiaustdiol	-1,51		Downregulated
Pro Ile (Hydroxyprolyl-Isoleucine)	-1,35	-1,11	Downregulated
Methoxamine	-1,37	-1,07	Downregulated
1-Octen-3-yl glucoside	2,46	1,62	Upregulated
Macrophyllic acid A	-1,52		Downregulated
Adouetine Y	-3,90		Downregulated
Homodihydrojasmone	1,68	1,00	Upregulated
Polysorbate 60	4,63	3,63	Upregulated
Arg Arg Gln (Oligopeptide)	-3,33		Downregulated
Pro Gln Lys (Oligopeptide)	-2,18		Downregulated
Lys Asn Lys (Oligopeptide)	-2,19		Downregulated
2-Phenylethanol glucuronide	-2,44		Downregulated
9-Octadecenoic acid, 18-fluoro-, (Z)- /Oleic acid, 18-fluoro-	1,31	1,21	Upregulated
alpha-Butyl-omega-hydroxypoly(oxyethylene)poly(oxypropylene)	-4,88	-1,04	Downregulated
(15:1)-Cardanol	1,25	1,07	Upregulated
(±)-1,4-Nonanediol diacetate	-1,79	-1,96	Downregulated
8E-Heptadecenedioic acid	-1,47	-1,02	Downregulated
Octadecyl fumarate	1,26		Upregulated
Alpha-Carboxy-delta-decalactone	-1,49	-1,61	Downregulated
Isoprenoid	1,39		Upregulated
4-Methyl-2-pentyloxazole	-1,52	-1,18	Downregulated
Longistylin A	1,04		Upregulated
Chrysanthemyl alcohol	2,23	1,48	Upregulated
PE (19:0/0:0)	1,00		Upregulated
Stearyl citrate	-2,15	-1,65	Downregulated

N-Methyl-2-pyridone-5-carboxamide (Nudifloramide)	-1,13	-1,04	Downregulated
6-Hydroxypentadecanedioic acid	-1,24		Downregulated
Nirvanol	-1,30	-1,14	Downregulated
Hydroxypelenolide	-1,05		Downregulated
Allyl propionate	-1,17		Downregulated
Methyl (7Z,9Z,9'Z)-6'-apo-y-caroten-6'-oate	1,15		Upregulated
PC(P-16:0/2:0)	1,03		Upregulated
N,N-Diethylglycine	-1,05		Downregulated
Carvyl acetate	-1,31		Downregulated
Octyl 2-furoate	-1,22	-1,22	Downregulated
Methylstyrylpyron	-1,96		Downregulated
Istamycin C1	-2,98		Downregulated
7-Oxo-11-dodecenoic acid	-4,42	-1,52	Downregulated
Styrene	-1,03	-1,41	Downregulated
5-Oxo-ETE-d7	-1,04		Downregulated
Tryptophyl-Lysine	-1,50		Downregulated
2-Benzoxazolol	-1,22		Downregulated
p-Coumaric acid	-1,11		Downregulated
(-)-Euphomine	-1,08		Downregulated
Glu Glu Asn	-3,19	-2,39	Downregulated
3,7-Dimethyl-3-octene-1,2,6,7-tetrol	-1,22		Downregulated
(4OH,8Z,t18:1) /// sphingosine	-1,03		Downregulated
Ser Arg Ile	-2,47		Downregulated
Dodecanoylcarnitine	-2,73		Downregulated
(3R,7R)-1,3,7-Octanetriol	-1,29		Downregulated

hexamethylene bisacetamide	1,06		Upregulated
6-Gingesulfonic acid	1,29		Upregulated
5-Aminoimidazole-4-carboxamide-1-β-D-ribofuranosyl 5'-monophosphate	1,34		Upregulated
2,3-di-0-hexanoyl-alpha-glucopyranose	1,04	1,38	Upregulated
6Z-8-Hydroxygeraniol 8-O-glucoside	1,58	1,40	Upregulated
Ro 31-6045	1,08		Upregulated
N2-Fructopyranosylarginine	1,29		Upregulated
Pymetrozine	-1,00		Downregulated
2'-Carboxy-4-[bis(2-chloroethyl)amino]-2-methylazobenzene	1,24		Upregulated
1-Methylpyrrolinium	1,30		Upregulated
Gly Thr Arg	1,37	1,42	Upregulated
Junionone	1,30		Upregulated
N-oleoyl methionine	-1,41		Downregulated
Octadecanoic acid, 18-fluoro-	1,24	1,25	Upregulated
Gly Gln Gln	-2,15		Downregulated
Oxprenolol	-1,33		Downregulated
5S-hydroxy-hexadecanoic acid	-2,62	-1,27	Downregulated
Polyethylene, oxidized	2,49	2,69	Upregulated
9-methyl-tridecanoic acid	-1,70	-1,80	Downregulated
Trans-7-octadecenoic acid; C18:1n-11	-1,83	-1,21	Downregulated
Triethyl citrate	-2,97		Downregulated
Glycidyl stearate	-1,22		Downregulated
MG(0:0/20:1(11Z)/0:0)	1,75		Upregulated
MG(0:0/22:1(13Z)/0:0)	1,18		Upregulated
Hexadecyl Acetyl Glycerol	-1,31		Downregulated

9-Riburonosyladenine	-2,73		Downregulated
MG(0:0/20:3(5Z,8Z,11Z)/0:0)	-1,26		Downregulated
Antioside	2,12		Upregulated
Avocadyne 4-acetate	-1,06		Downregulated
6Z-Tetradecenyl acetate	-2,39	-1,69	Downregulated
L-Valine	-2,04	-1,25	Downregulated
(10S)-Juvenile hormone III diol	-1,08		Downregulated
8-(5-hexyl-furan-2-yl)-octanoic acid	-2,68	-1,23	Downregulated
Val Val Arg	-2,33	-1,10	Downregulated
1,3-Diisopropylbenzene	-2,26	-2,05	Downregulated
Thiopental	1,47	-1,46	Downregulated
Ethyl 2-aminobenzoate	-1,62	-1,30	Downregulated
(5Z,8Z)-1,5,8-Heptadecatriene	-1,55	-2,08	Downregulated
Arg Leu Ala	2,61	-1,31	Downregulated
5-Methylheptan-2-one	1,81	-1,44	Downregulated
Tanacetol B	-1,70	-1,21	Downregulated
Neostigmine	-1,84	-1,03	Downregulated
6Z-Tetradecenyl acetate	-3,07	-1,69	Downregulated
3-methoxy Prostaglandin F1α	-1,29	-1,04	Downregulated
13-Hydroxy-9-methoxy-10-oxo-11-octadecenoic acid	-1,11	-1,40	Downregulated
Indanestrol	-2,39	-1,64	Downregulated
Gln Glu Glu		-1,93	Downregulated
Ethiofencarb		-1,48	Downregulated
2-Benzoxazolol		-1,18	Downregulated
Pro Asp Asn		-2,91	Downregulated

(3S,5R,6R,7E)-3,5,6-Trihydroxy-7-megastigmen-9-one	-1,12	Downregulated
C15:1n-1	-1,35	Downregulated
Patchoula-2,4-diene	-1,49	Downregulated
3α,12α-Dihydroxy-5β-cholest-24-en-26-oic acid	-1,02	Downregulated
PG(22:0/0:0)	-1,32	Downregulated
ω-3 Arachidonic Acid-d8	-1,99	Downregulated
Isosorbide-2-glucuronide	-4,59	Downregulated
Piperocaine	-1,48	Downregulated
7-Tridecenoic acid; C13:1n-6	-1,16	Downregulated
Trans-5,6-Dihydro-5,6-dihydroxy-7,12-dimethylbenz[a]anthracene	-1,37	Downregulated
Acetylpseudotropine	-1,51	Downregulated
Polysorbate 20	-1,97	Downregulated
Pirbuterol	-1,20	Downregulated
Ser Gly Arg	1,49	Upregulated
7,7-Difluoro-8Z-dodecenyl acetate	-1,30	Downregulated
Ornaline	-1,33	Downregulated
Gamma-linolenyl carnitine	-1,32	Downregulated
Bornyl isovalerate	-1,44	Downregulated
Indospicine	-1,87	Downregulated
Val Arg Ala	-1,84	Downregulated
N4-Phosphoagmatine	-1,01	Downregulated
beta-Farnesene	-1,42	Downregulated
Mesitylene	-1,24	Downregulated
2,4-Diphenyl-1-butene	-1,04	Downregulated
Pyruvophenone	-1,30	Downregulated

Thr Thr Arg	1,17	Upregulated
Fukinone	-1,51	Downregulated
D-erythro-Sphingosine C-15	-1,36	Downregulated
Norecasantalic acid	-1,44	Downregulated

Table S2. Results from pathway analysis

Pathway	Total	Expected	Hits	Raw p	-log10(p)	Holm adjust	FDR	Impact
Biosynthesis of unsaturated fatty acids	36	0.50286	4	**9.3291e-05**	4.0302	**0.0074633**	**0.0074633**	0
Glycerophospholipid metabolism	36	0.50286	1	**0.012535**	1.9019	0.99024	0.50139	0.19552
Fatty acid biosynthesis	47	0.65651	3	**0.025767**	1.5889	1	0.60806	0.01473
Ether lipid metabolism	20	0.27937	2	**0.030403**	1.5171	1	0.60806	0
Linoleic acid metabolism	5	0.069841	1	**0.068001**	1.1675	1	0.99112	1
Glycine, serine and threonine metabolism	33	0.46095	2	**0.075766**	1.1205	1	0.99112	0.25981
Arginine and proline metabolism	36	0.50286	2	**0.08817**	1.0547	1	0.99112	0.02442
Valine, leucine and isoleucine biosynthesis	8	0.11175	1	0.10666	972	1	0.99112	0
Ascorbate and aldarate metabolism	9	0.12571	1	0.1192	0.92372	1	0.99112	0
Arachidonic acid metabolism	44	0.6146	2	0.12389	0.90696	1	0.99112	0.29133
Nicotinate and nicotinamide metabolism	15	0.20952	1	0.191	0.71898	1	1	0
Glycosylphosphatidylinositol (GPI)-anchor biosynthesis	15	0.20952	1	0.191	0.71898	1	1	0.00639
Retinol metabolism	17	0.23746	1	0.21366	0.67028	1	1	0
Pentose and glucuronate interconversions	19	0.2654	1	0.23572	0.62761	1	1	0.10843

Pantothenate and CoA biosynthesis	20	0.27937	1	0.24652	0.60814	1	1	0
Lipoic acid metabolism	28	0.39111	1	0.32787	0.48429	1	1	0.0017
Glutathione metabolism	28	0.39111	1	0.32787	0.48429	1	1	0.08873
Porphyrin metabolism	31	0.43302	1	0.35616	0.44835	1	1	0
Sphingolipid metabolism	32	0.44698	1	0.36533	0.43731	1	1	0.06191
Glyoxylate and dicarboxylate metabolism	32	0.44698	1	0.36533	0.43731	1	1	0.10582
Cysteine and methionine metabolism	33	0.46095	1	0.37438	0.42668	1	1	0.10446
Fatty acid elongation	39	0.54476	1	0.42615	0.37044	1	1	0
Fatty acid degradation	39	0.54476	1	0.42615	0.37044	1	1	0
Valine, leucine and isoleucine degradation	40	0.55873	1	0.43436	0.36215	1	1	0
Primary bile acid biosynthesis	46	0.64254	1	0.48137	0.31752	1	1	0.00758
Metabolism of xenobiotics by cytochrome P450	68	0.94984	1	0.6238	0.20495	1	1	0
Purine metabolism	70	0.97778	1	0.63471	0.19743	1	1	0.07298
